# Delayed-Onset Postsurgical Myofascial Pain Following Axillary Artery Cannulation: A Case Report

**DOI:** 10.7759/cureus.91853

**Published:** 2025-09-08

**Authors:** Ikuyo Watanabe, Katsuyuki Moriwaki, Rumiko Hachisuka, Ryuji Nakamura, Yasuo M Tsutsumi

**Affiliations:** 1 Department of Anesthesiology, Hiroshima University Hospital, Hiroshima, JPN; 2 Department of Anesthesiology, Hiroshima Hiramatsu Hospital, Hiroshima, JPN; 3 Department of Anesthesiology and Critical Care, Graduate School of Biomedical and Health Sciences, Hiroshima University, Hiroshima, JPN

**Keywords:** axillary artery cannulation, chronic postsurgical pain, myofascial pain syndrome, pectoral nerve block, ultrasound-guided trigger point injection

## Abstract

Chronic postsurgical pain (CPSP) is often attributed to neuropathic, genetic, or psychosocial factors, whereas musculoskeletal causes, such as myofascial pain syndrome (MPS), are frequently overlooked. We report a case of delayed-onset MPS as a musculoskeletal manifestation of CPSP following axillary artery cannulation, aiming to raise awareness of this potential etiology. A 48-year-old man developed persistent right anterior chest pain one year after total arch replacement surgery. Physical examination revealed postural asymmetry, atrophy of the right pectoralis major muscle, mechanical allodynia, and myofascial trigger points (MTrPs) near the cannulation site and scapula. Ultrasonography demonstrated the disrupted architecture of the pectoralis major and adjacent connective tissue. Based on clinical and sonographic findings, MPS was diagnosed. Treatment included pregabalin, ultrasound-guided trigger point injections (TPIs) with levobupivacaine and triamcinolone, and type II pectoral nerve blocks. Five biweekly sessions led to significant pain relief, resolution of allodynia, and improved posture. This case highlights the importance of recognizing delayed-onset MPS as a musculoskeletal manifestation of CPSP following axillary artery cannulation, likely related to localized muscle trauma and pectoral nerve injury, and supports the value of multimodal management.

## Introduction

Chronic postsurgical pain (CPSP) is defined in the International Classification of Diseases 11th Revision (ICD-11) as pain that begins or increases after surgery, persists for at least three months, and cannot be explained by other conditions such as infection or malignancy [1]. CPSP is reported to occur in up to 50% of patients following common surgical procedures, with severe pain affecting up to 10% of patients [2]. Moreover, CPSP is relatively common after cardiac surgery; persistent pain is reported in up to 43% of patients at three months after coronary artery bypass grafting or thoracotomy and in 17% of patients even after two years [3]. Recent studies have emphasized that CPSP is a multifactorial condition involving neuropathic mechanisms (e.g., nerve injury), central and peripheral sensitization, inflammatory immune responses, and psychosocial factors [2,4]. In addition, although the number of reports remains limited and it is not yet widely recognized as a common condition, myofascial pain syndrome (MPS) has been identified as a potential contributor to persistent postsurgical pain [5-10]. MPS is a musculoskeletal pain disorder characterized by the presence of myofascial trigger points (MTrPs), which are hyperirritable spots in skeletal muscle associated with palpable nodules [11,12]. These trigger points can cause local tenderness, referred pain, and functional limitation [11,12].

In the present case, the patient developed persistent and refractory pain in the right subclavian region 12 months after undergoing total arch replacement for Stanford type A aortic dissection. Axillary artery cannulation is a standard approach for antegrade aortic and cerebral perfusion in such surgeries [13,14] and is also used in procedures such as extracorporeal membrane oxygenation (ECMO) and transcatheter aortic valve implantation (TAVI) [14]. This technique involves dissection of the pectoralis major and minor muscles and careful exposure of the axillary artery adjacent to the brachial plexus, which poses a risk of nerve or muscle injury [14]. To the best of our knowledge, this is the first report demonstrating that CPSP following axillary artery cannulation can be attributed to MPS, which was most likely secondary to the nerve or muscle injury.

## Case presentation

A 48-year-old man (height: 168 cm, weight: 52 kg) with a history of bronchial asthma and insomnia underwent emergency total arch replacement for acute Stanford type A aortic dissection. Surgery was performed via median sternotomy using arterial cannulation of the right axillary and left femoral arteries. Postoperatively, the patient developed ischemic colitis, which resolved with conservative management, and was discharged on postoperative day 12. Two months later, the patient underwent sigmoid colectomy for bowel obstruction because of adhesions after colitis.

Since the aortic arch replacement, the patient had experienced difficulty elevating his right shoulder, which gradually improved over approximately 10 months with physical therapy. However, approximately 12 months postoperatively, the patient began to experience discomfort and pain in the right anterior chest, accompanied by progressive rightward trunk deviation. Despite ongoing rehabilitation and oral loxoprofen treatment, the patient's symptoms persisted. The patient was referred to our department 17 months after surgery.

**Figure 1:**
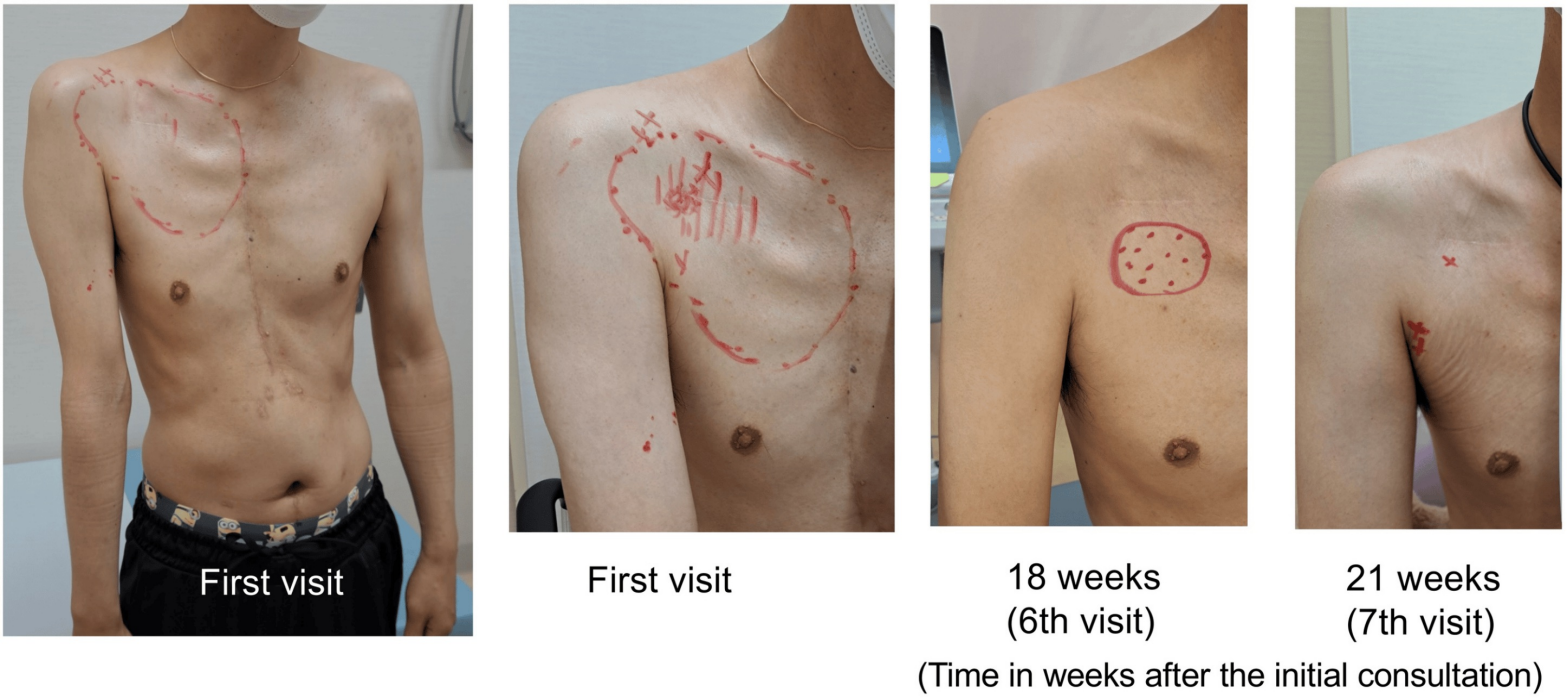
Initial postural deviation and the temporal-spatial progression of mechanical allodynia and tenderness The patient showed limited right shoulder elevation, right trunk deviation, and atrophy of the right pectoralis major at the first visit. Tender points, including MTrPs, were found in the right pectoralis major and around the axillary artery cannulation scar (marked "X"). Areas of allodynia/tenderness (red), pronounced tenderness (red slashes), and tender points (red dots) are indicated. By week 21, allodynia and tenderness had resolved except for a few residual tender points. To assess mechanical allodynia, dynamic mechanical stimulation was applied by lightly stroking the skin with a cotton swab over a distance of a few centimeters. MTrPs: myofascial trigger points

At the initial visit, the patient reported persistent pain beneath the right clavicle at the surgical scar site, with a numerical rating scale (NRS) score of 8. He reported a squeezing sensation extending from the right axilla to the lateral chest wall, which was slightly relieved by applying pressure to the area. Physical examination revealed limited right shoulder elevation, right trunk deviation, and visible atrophy of the right pectoralis major (Figure 1). Although the pain compromised his sleep and psychological health, he remained employed and managed to preserve his basic home functioning.

**Figure 2:**
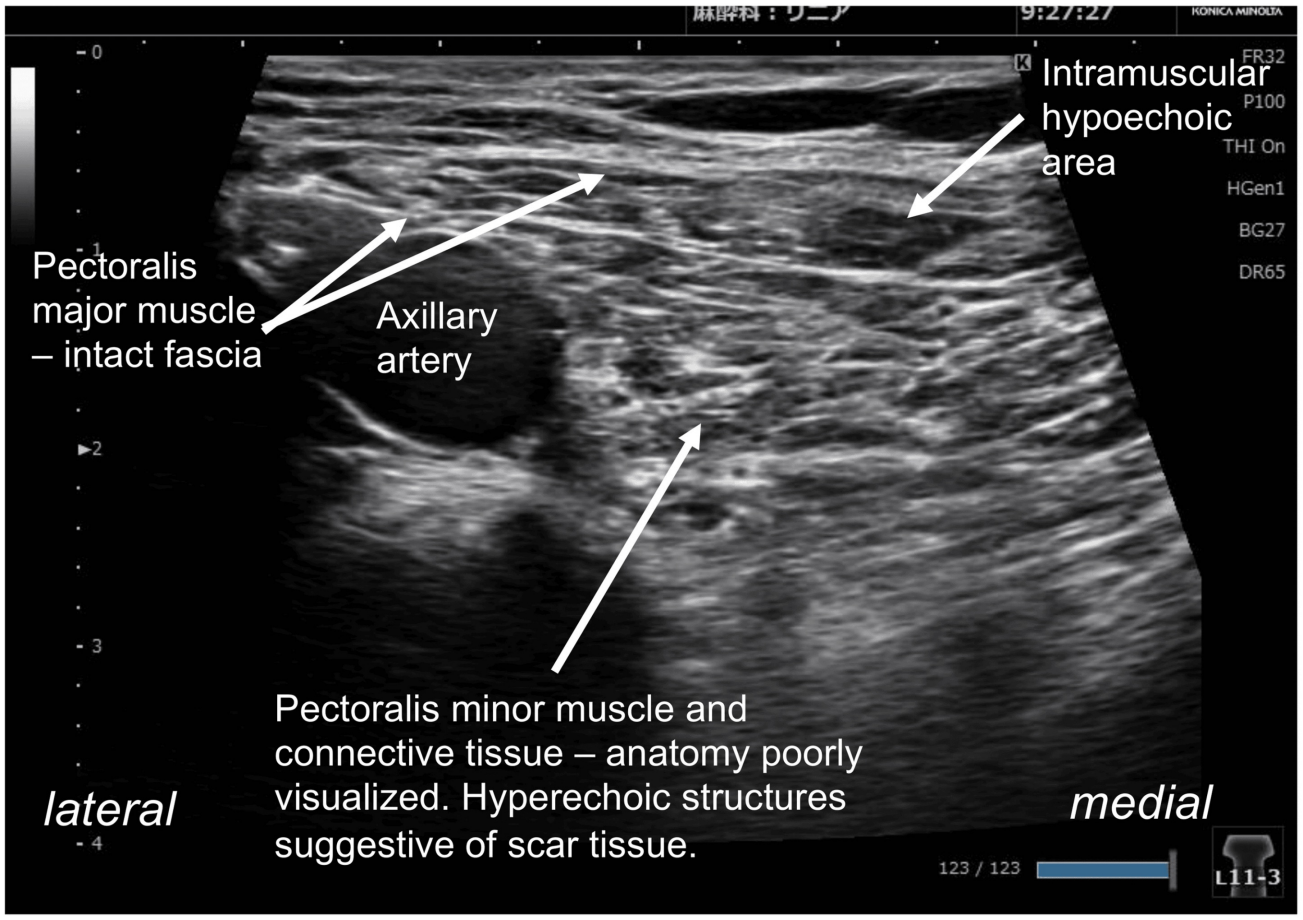
Ultrasonographic findings near the right axillary artery Ultrasound image showing a mixture of hyperechoic and hypoechoic regions around the right axillary artery. These findings indicate localized structural abnormalities in the adjacent pectoralis major and minor muscles.

Sensory examination revealed mechanical allodynia to light touch in the right anterior chest (Figure 1), accompanied by tenderness, suggesting a reduced pain threshold. Motor and sensory functions in the right upper extremity were intact, and no signs of autonomic dysfunction, such as abnormal sweating or edema, were observed. The bilateral grip strength was maintained at 26 kg. MTrPs were identified in the right pectoralis major muscle, just cranial to the scar from the right axillary artery cannulation (Figure 1), along with multiple tender points in the right infraspinatus muscle. Thermographic imaging revealed no abnormalities, and laboratory tests showed no signs of inflammation, including leukocytosis or elevated C-reactive protein levels. Ultrasonography revealed mixed hyperechoic and hypoechoic areas near the right axillary artery, suggesting localized structural and muscle abnormalities (Figure 2). No pain or sensory disturbance was observed around the sternotomy site.

The patient was initially prescribed low-dose pregabalin (25 mg/day); however, the medication was discontinued because of worsening asthma symptoms. After asthma stabilization, pregabalin was resumed at 150 mg/day (75 mg twice daily) three months after the initial consultation. In addition, a pectoral nerve block type II (PECS II block) (10 mL of 0.25% levobupivacaine) and ultrasound-guided trigger point injections (TPIs) using 10 mL of 0.25% levobupivacaine and 10 mg of triamcinolone were administered to the tender points beneath the right clavicle and multiple tender points in the right infraspinatus muscle. The pressure applied during the injections often elicited jump signs. One week after the initial intervention, the patient's NRS score decreased to 3. Five PECS II block and TPI sessions were performed at intervals longer than two weeks, spanning a total of 18 weeks. Although a transient flare-up of pain (NRS score of 8) occurred during the winter, the pain subsequently decreased to an NRS score of 5 or below by week 21. Allodynia gradually resolved (Figure 1), and the patient's posture returned to normal. Thereafter, his pain remained well-controlled with oral pregabalin alone.

## Discussion

Suspected right pectoral nerve injury

In this case, the patient experienced impaired elevation of the right shoulder after surgery, and right trunk deviation and visible atrophy of the right pectoralis major muscle were noted during the initial consultation. Based on the clinical course and physical findings, injury to the lateral and/or medial pectoral nerves, which is potentially associated with axillary artery cannulation, was suspected to be the primary cause.

The lateral and medial pectoral nerves, which arise from the brachial plexus and innervate the pectoralis muscles, can be injured by traction, compression, or surgical manipulation during axillary artery cannulation [15,16]. A computed tomography-based study of patients undergoing this procedure reported long-term atrophy of the pectoralis major and minor muscles, suggesting that intraoperative trauma can result in persistent morphological and functional impairments [16]. These findings support the likelihood that pectoral nerve injury contributed to muscle atrophy and dysfunction in our patient.

The lateral pectoral nerve (LPN) primarily innervates the clavicular and upper sternal portions of the pectoralis major muscle and plays a critical role in shoulder flexion and elevation [15]. Severe injury to the LPN can result in denervation of most pectoralis major muscles [15]. Our patient's impaired shoulder movement suggests possible LPN involvement, which is consistent with reports of anterior chest pain and shoulder elevation impairment following LPN injury [15,17]. In addition, the medial pectoral nerve, which innervates the lower pectoralis major and minor muscles, may have been injured, contributing to scapular dysfunction and postural deviation [15].

Postoperative MPS

MTrPs were identified in the pectoralis major muscle near the axillary artery cannulation site and the ipsilateral infraspinatus muscle, and the patient's symptoms were consistent with MPS [11,12]. Recently, Steen et al. proposed five essential clinical signs for the diagnosis of MPS: the presence of one or more trigger points, pain reproduction with pressure, a typically referred pain pattern, a palpable taut band, and a local twitch response, building on Simons' criteria [12]. Our patient fulfilled all the diagnostic criteria.

In our case, MPS became clinically apparent one year after surgery. In a prospective study of women undergoing breast cancer surgery, 44.8% developed MPS within one year [6]. Notably, the most active MTrPs appeared within the first six months but were not always present immediately after surgery, supporting the possibility of delayed-onset MPS [6]. Such delayed-onset MTrPs have also been reported after mastectomy in a case series [7].

MTrPs are thought to result from localized ischemia and sustained muscle contraction owing to calcium dysregulation, a state referred to as the "energy crisis" [11,12]. Acute muscle trauma, including surgical injury or contusion, may trigger this process, with pain and dysfunction often persisting long after the resolution of the initial tissue injury [12]. In our patient, axillary cannulation probably caused trauma to the pectoralis muscle and/or pectoral nerves, leading to a delayed onset of MTrPs. The patient with delayed-onset MPS responded well to TPIs and PECS II blocks, underscoring the importance of recognizing and treating MPS to achieve significant symptom relief.

Reversible sensory disturbances

Recent advances suggest that MPS involves peripheral nociceptive mechanisms, central sensitization, and features of nociplastic pain [18]. Sensory abnormalities, such as allodynia, hyperalgesia, and tactile hypoesthesia, have been reported in patients with MPS, and many of these are reversible with appropriate treatment [18,19]. In the present case, mechanical allodynia to light touch was observed in the area surrounding the trigger point, which rapidly resolved following TPIs. This clinical course suggests that ongoing peripheral input from MTrPs may contribute to the maintenance of central sensitization and that such sensitization can be reversed by appropriate interventions. On the other hand, tactile hypoesthesia, commonly observed in MPS, has been reported to diminish or disappear following successful treatment with TPIs [19]. These reversible changes in the spatial distribution of allodynia and hypoesthesia may reflect dynamic alterations in central pain processing and serve as useful clinical indicators [19,20].

Reduced pressure pain thresholds (PPTs) have also been reported in patients with persistent postoperative pain, including those who have undergone breast cancer surgery, supporting the role of altered central pain processing [9]. In this case, the pain reproducibly elicited by light pressure diminished along with the spatial extent of tactile hypersensitivity after five sessions of TPIs and the PECS II block. These changes suggest that, in addition to allodynia, monitoring the distribution of reduced PPT and allodynia may provide useful clinical insights into the progression and resolution of MPS-related central sensitization.

TPI and PECS II block

Current treatment options for MPS include local anesthetic injections, dry needling, physical therapy, postural correction, and emerging modalities such as botulinum toxin and platelet-rich plasma injections. However, the optimal therapeutic strategy remains unclear [12]. High-quality evidence remains limited, particularly in the context of postoperative MPS. In this case, TPIs using levobupivacaine and triamcinolone effectively resolved the MTrPs and alleviated the pain. The addition of triamcinolone may have contributed to the suppression of local inflammation in MTrPs.

Regarding ultrasound-guided techniques, a previous case report described subacute postoperative abdominal wall pain following laparoscopic liver resection that was diagnosed as MPS based on the ultrasonographic identification of a trigger point in the internal oblique muscle and was successfully treated with TPIs, resulting in rapid symptom relief [10]. In the present case, delayed-onset myofascial pain responded well to a combination of ultrasound-guided TPIs and PECS II blocks. The PECS block likely contributes to symptom relief by attenuating hypersensitivity mediated by the lateral and medial pectoral nerves, which aligns with previous reports of its effectiveness in chest wall pain associated with LPN injury [17].

Pregabalin was also administered as an adjunct pharmacotherapy, and the synergistic effect of pharmacological and interventional treatments likely contributed to the overall clinical improvement. This case highlights the potential value of a multimodal approach combining TPI, PECS II block, and pharmacotherapy for managing delayed-onset MPS following surgical intervention.

Limitations

A key limitation of this study is the absence of electrodiagnostic evidence directly linking suspected pectoral nerve injury to MTrP development. Although clinical findings suggested a relationship between nerve injury and MPS, possible mechanisms such as neuromuscular junction abnormalities from nerve regeneration or secondary MPS due to disuse atrophy remain hypothetical without objective data.

## Conclusions

This case highlights the importance of considering delayed-onset MPS as a musculoskeletal cause of CPSP following axillary artery cannulation. Careful physical examination and sonographic assessment facilitated accurate diagnosis, and a multimodal approach including ultrasound-guided trigger point injections and PECS II blocks led to significant symptom relief and functional recovery. Increased awareness of postoperative MPS may promote timely intervention and improved patient outcomes.

## Appendices

Patient perspective

I had been suffering from pain for a long time, so I was relieved when the cause was finally identified. As the treatment gradually eased my pain and made it easier to move, I felt truly happy.
